# Vitamin D status and bone mineral density in the Chinese population: a review

**DOI:** 10.1007/s11657-016-0265-4

**Published:** 2016-03-30

**Authors:** P. W. Man, I. M. van der Meer, P. Lips, B. J. C. Middelkoop

**Affiliations:** 1 Department of Public Health and Primary Care, Leiden University Medical Center, PO Box 9600, 2300 RC Leiden, The Netherlands; 2 Department of Epidemiology, Municipal Health Service Haaglanden, The Hague, The Netherlands; 3 Department of Internal Medicine, Endocrine Section, VU University Medical Center, Amsterdam, The Netherlands

**Keywords:** Bone mineral density, Chinese, Vitamin D status

## Abstract

**Summary:**

Low vitamin D status is associated with low bone mass which, in turn, is an important predictor of fracture. However, data on this relationship in non-Caucasian populations are scarce. This review shows such an association in the Chinese population in five of the 11 included studies.

**Introduction:**

In the elderly population, the serum 25-hydroxyvitamin D [25(OH)D] concentration is often inadequate. This may cause a lower bone mineral density (BMD), which is an important predictor of fracture. It is estimated that by 2050 more than half of all hip fractures worldwide will occur in Asia. However, data on the relationship between vitamin D status and BMD in a non-Caucasian population are scarce. Therefore, this study reviews the literature on the relationship between serum 25(OH)D and BMD in the Chinese population.

**Methods:**

A search was made in PubMed, EMBASE, Web of Science and Cochrane Library (up to December 2014) to identify relevant studies using the terms vitamin D status, bone mineral density, and Chinese.

**Results:**

Of the 293 studies identified, 11 fulfilled the inclusion and exclusion criteria and were analyzed. Mean serum 25(OH)D concentrations ranged from 29-82 nmol/L. In 5 of the 11 studies, an association was found between vitamin D status and BMD in the Chinese population.

**Conclusion:**

The evidence for a relationship between the serum 25(OH)D concentration and BMD in the middle-aged and elderly Chinese population living in Asia appears to be limited and inconsistent.

## Introduction

Vitamin D is important for the development, growth, and maintenance of a healthy skeleton. Low vitamin D status is suggested to contribute to bone loss and a low bone mineral density (BMD) is an important predictor of osteoporotic fractures. Fractures cause impaired quality of life [1], are associated with increased mortality [2] and are accompanied by high cost. Bone loss also occurs with aging and the prevalence of osteoporosis is expected to increase as life expectancy rises. As a result of a large aging Asian population, it is estimated that by 2050 more than 50 % of all hip fractures worldwide will occur in Asia [3]. For example, in Hong Kong, the incidence of hip fractures in older men and women more than doubled between 1966 and 1985 [4]. In Beijing, the incidence rate of hip fracture showed a greater increase from 2002 to 2006 than from 1990 to 1992 [5]. Rapidly increasing industrialization and urbanization and reduction of weight-bearing activities are also associated with an increase in the incidence of fractures in various areas in Asia [6]. By contrast, during the past decade a decline has been reported in the incidence of hip fractures in the more developed regions of Asia (e.g., Hong Kong). An increase in BMD levels, and a healthier lifestyle with more physical activity and higher vitamin D status, may explain this decline in the incidence of osteoporosis and fractures [7].

Vitamin D_3_, or cholecalciferol, is primarily produced in the skin from 7-dehydrocholesterol when exposed to the ultraviolet light of the sun (UVB 290–315 nm). It can also be obtained from food, especially fatty fish such as salmon, mackerel, eel, and herring and may also be added to food as fortification, e.g., to margarine or milk. In the liver vitamin D_3_ is rapidly hydroxylated into 25-hydroxyvitamin D [25(OH)D], which has low biological activity but is the major circulating metabolite. It is further hydroxylated in the kidney into 1,25-dihydroxyvitamin D [1,25(OH)_2_D], the active form of vitamin D. This latter hydroxylation is stimulated by parathyroid hormone (PTH). The main effect of 1,25(OH)_2_D is to stimulate the intestinal absorption of calcium and phosphate for the mineralization of the skeleton and for neuromuscular action.

The vitamin D status is usually assessed by measuring the serum 25(OH)D concentration. Insufficient exposure to sunlight, covering of the skin, skin pigmentation, a diet that is low in vitamin D and calcium, and advanced age may negatively affect vitamin D status. Consequences of severe vitamin D deficiency include rickets in children and osteomalacia in adults. In addition, it may lead to secondary hyperparathyroidism, high bone turnover, bone loss, mineralization defects and it predisposes to fractures. Muscle weakness is another consequence of low 25(OH)D levels.

Studies among Caucasian populations reported the relationship between vitamin D status and BMD, with evidence of a positive association [8, 9]. By contrast, low 25(OH)D concentrations were not correlated with low BMD among Black individuals [10] and among Black or Hispanic men [11]. These findings suggest that the relationship between 25(OH)D and BMD may differ by race and ethnicity. There are various reasons why the relationship between 25(OH)D and BMD might be different in the Chinese population compared to the Caucasian population. First, genetic factors, for example variants of vitamin D metabolizing enzymes CYP24A1 (24-hydroxylase), CYP2R1 (25-hydroxylase), vitamin D binding protein (DBP), and DHCR7 (7-dehydrocholesterol reductase) can contribute to variation in 25(OH)D concentrations [12, 13]. Second, the average dietary calcium intake in the Chinese population is low, leading to secondary hyperparathyroidism and increased turnover of vitamin D metabolites. This may also affect the relationship between 25(OH)D and BMD. In addition, some lifestyle factors, e.g., (outdoor) physical activity, may also influence vitamin D status and BMD, and other lifestyle factors (e.g., behavior in the sun) may differ between races as, for example, the Chinese population tend to have a sun-avoiding behavior.

Information on the relationship between 25(OH)D and BMD in the Chinese population is scarce but is relevant in view of the high occurrence of fractures in Asia. In addition, insight into the relationship may help formulate recommendations for the use of vitamin D supplementation to prevent osteoporosis.

Therefore, the present study reviews the literature on the relationship between vitamin D status and BMD in the middle-aged and older Chinese population. The influence of the latitude of the area, season, gender, and age on serum 25(OH)D levels is also examined.

## Methods

The following electronic databases were searched up to December 16 2014: PubMed, EMBASE, Web of Science, and Cochrane Library. Keywords included vitamin D status, bone mineral density, and Chinese. The search strategy included both truncated free-text and MeSH terms and was adjusted according to different databases. The complete search strategy is presented in Appendix A. Restriction was made to full-text articles and abstracts in the English and Dutch language. Chinese full-text articles with an English abstract were translated into English. Publications were eligible when reporting an association between serum 25(OH)D concentrations and BMD or bone mineral content (BMC) in the Chinese population.

## Data extraction

Extracted from the included studies were the following: (1) mean (±SD) serum 25(OH)D concentrations (nmol/L), (2) percentage of subjects with serum 25(OH)D levels ≤25 (or 37.5) nmol/L, between 25 and 50 nmol/L, between 50 and 75 nmol/L, and ≥75 nmol/L, (3) mean (±SD) age of the subjects (years), (4) gender, (5) latitude of the area where the study was performed (° N), and (6) associations between serum 25(OH)D levels and BMD or BMC.

All serum 25(OH)D concentrations were converted to nmol/L (1 ng/ml = 2.496 nmol/L).

## Results

**Fig. 1:**
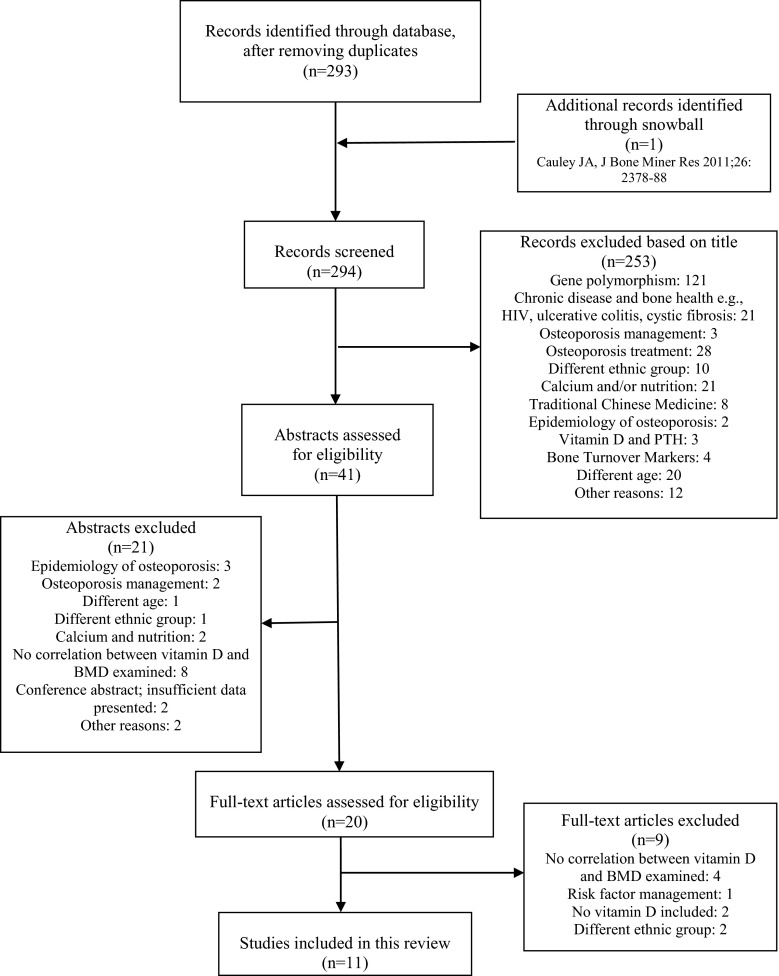
Summary of the selection strategy to include articles for the present review

Figure 1 presents the results of the literature search; reasons for exclusion based on title, abstract, or full text are given. The characteristics of the selected studies are outlined in Table 1.

**Table 1:** Description of studies investigating the relationship between the serum 25(OH)D concentration and BMD in the Chinese population

Reference	Latitude	Population	Age of subjects (years, mean ± SD)	s-25(OH)D (nmol/L, mean ± SD)	Proportion of subjects with s-25(OH)D (nmol/L)	Association between serum 25(OH)D concentration and BMD	Adjusted variables
Chee et al. (Kuala Lumpur, Malaysia) 2010 [16]	3° N	Malaysian Chinese women, postmenopausal (*n* = 178)	59.7 ± 5.0	60.4 ± 15.6	<50: 50.6 %	No significant correlation between 25(OH)D and lumbar spine BMD (*r* = 0.035) and between 25(OH)D and femoral neck BMD (*r* = −0.004). No significant difference in the vitamin D status of subjects with osteoporosis, osteopenia and normal BMD at the spine and femoral neck	
Woo et al. (Hong Kong, China) 1994 [22]	22° N	Hong Kong Chinese women (*w*, *n* = 213), men (*m*, *n* = 104)	*w*: 76.0 ± 7.7; *m*: 74.3 ± 6.3	*w*: 66.8 ± 25.5; *m*: 69.3 ± 20.3		25(OH)D is not significantly correlated with BMD at lumbar spine (*w*: *r* = −0.18; *m*: *r* = -0.08) and at three regions in the left hip: neck (*w*: *r* = -0.02; *m*: *r* = 0.19), trochanter (*w*: *r* = −0.03; *m*: *r* = 0.13), and Ward’s triangle (*w*: *r* = 0.12; *m*: *r* = 0.12)	
Wat et al. (Hong Kong, China) 2007 [21]	22° N	Hong Kong Chinese women (*w*, *n* = 179), men (*m*, *n* = 203)	69.6 ± 9.0	70.8 ± 27.0 (difference summer vs. winter samples: NS)	<25: 0.8 %; 25–50: 21.7 %; 50–75: 40.3 %; >75: 37.2 %	Subjects with 25(OH)D < 75 nmol/L had significantly lower BMD at both lumbar spine and total hip, even after adjusting for sex and age	Sex, age
Chan et al. (Hong Kong, China) 2011 [14]	22° N	Hong Kong Chinese men (*m-cs*, *n* = 939 cross-sectional; *m-fu*, *n* = 712 follow-up)	72.8 ± 5.1 (*m*-*cs*); 72.2 ± 4.8 (*m*-*fu*)	77.9 ± 20.5 (*m*-*cs*); 78.2 ± 20.5 (*m*-*fu*)	<50: 5.9 %; 50–75: 41.5 %; >75: 52.6 %	After adjustment for potential confounding factors a higher level of s-25(OH)D was associated with a higher BMD at spine (*B* = 0.001, *p* = 0.026), femoral neck (*B* = 0.0004, *p* = 0.015) and total hip (*B* = 0.0004, *p* = 0.022) at baseline. No association between serum 25(OH)D and BMD change after 4-year follow-up at all sites	Age, BMI, education, physical activity, quality of diet, alcohol use
Tsai et al. (Taipei, Taiwan) 1997 [19]	25° N	Chinese women (*n* = 262)	40–53 (*n* = 67, premenopausal);46–72 (*n* = 195, postmenopausal)	76.8 ± 20.5 (all women; *n* = 138, summer; *n* = 124, winter). Subset of 54 women (aged 53–65 years): 81.8 ± 19.0 (summer) 71.3 ± 20.8 (winter), *p *<0.001	<37.5: 2.5 %	No association between 25(OH)D concentration and BMD at lumbar spine (L2–L4) and proximal femur	Age, body weight, height
Li et al. (Changsha, China) 2014 [17]	28° N	Chinese women, postmenopausal (*n* = 578)	62.2 ± 6.1	43.5 ± 14.3 (summer)	<50: 72.1 %	Positive correlation between 25(OH)D and lumbar spine (L1–L4, *r* = 0.094, *p* < 0.05), femoral neck (*r* = 0.080, *p* < 0.05), and total hip BMD (*r* = 0.103, *p* < 0.05). After adjustment for potential confounding factors: lumbar spine (L1–L4, *r* = 0.155, *p* < 0.001); femoral neck (*r* = 0.143, *p* < 0.01), and total hip (*r* = 0.166, *p* < 0.001)	Age, BMI, serum creatinine levels, calcium supplementation
Zhen et al. (Lanzhou, China) 2014 [24]	36° N	Chinese women (*w*, *n* = 7136), men (*m*, *n* = 2902)	40–75	*w*: 39.2 ± 17.8; *m*: 45.3 ± 15.7	<50: 75.2 %	No correlation between serum 25(OH)D levels and BMD values measured by QUS	Age, gender, BMI, waist circumference, diabetes, dyslipidemia, hypertension, physical activity, smoking, alcohol drinking, coffee drinking, tea drinking, self-reported coronary heart disease, self-reported stroke, serum and urine phosphorus and calcium levels, sun exposure time
Liu et al. (Beijing, China) 2010 [18]	40° N	Chinese women, postmenopausal (n = 400)	60–85 (median age: 67.8)	36.0 ± 14.6	<50: 84.2 %; >75: 3.8 %	Positive significant correlation between 25(OH)D and BMD at total body (*r* = 0.17, *p* < 0.01) and proximal femur (*r* = 0.18, *p* < 0.01)	Age, height, weight, season of blood sampling
Zhao et al. (Beijing, China) 2011 [20]	40° N	Chinese women, postmenopausal (*n* = 1724)	64.1 ± 9.2	33.0 ± 13.5	<50: 89.7 %; >75: 0.6 %	No significant correlation between 25(OH)D and BMD (partial correlation analysis): lumbar spine (*r* = −0.117, *p* = 0.053); femoral neck (*r* = −0.031, *p* = 0.208), and total hip (*r* = 0.053, *p* = 0.073)	Age, height, weight, years since menopause
Yan et al. (Shenyang, China) 2003 [23]	42° N	Chinese women (*w*, *n* = 110), men (*m*, *n* = 108)	*w*: 65.2 ± 3.7; *m*: 67.9 ± 3.6	*w*: 30.9 ± 13.5; *m*: 27.1 ± 11.5 (*p* = 0.03) (late winter samples)	<25: 39.1 % *w* (*n* = 110), 52.8 % *m* (*n* = 108), *p* < 0.05	No significant association between plasma 25(OH)D and BMC at any site	Age, sex, bone area, weight, height
Zhou et al. (Shenyang, China) 2011 [15]	42° N	Chinese women (*w*, *n* = 54), men (*m*, *n* = 46)	*w*: 64.6 ± 3.8; *m*: 66.8 ± 4.7 (baseline)	31.0 ± 12.3 (baseline)	<25: 40 %; >75: 0 %	After 5 years: negative correlation between bone loss rate at femoral neck and 25(OH)D concentration at baseline (*r* = −0.27, *p* = 0.02, personal communication with the author). Bone loss rate at femoral neck was 103 % higher for subjects with 25(OH)D concentrations <25 nmol/L compared to subjects with 25(OH)D concentrations >25 nmol/L	Age, weight, height

*BMD* bone mineral density, *BMC* bone mineral content, *BMI* body mass index, *PTH* parathyroid hormone, *QUS* quantitative ultrasound, *NS* not significant

Of the 11 included studies most had a cross-sectional design. Two studies combined a cross-sectional design with a 4- and 5-year follow-up study [14, 15]. Five studies included only women [16–20], five studies included both men and women [15, 21–24], and one study included only men [14]. The mean age of the subjects in most of the studies ranged from 60 to 76 years. The studies performed in Lanzhou [24] and Taipei [19] differed from the other studies in that they included men and women from age 40 years, and included both premenopausal and postmenopausal women, respectively.

Four studies recruited participants randomly [17, 18, 20, 24], and one study reported response rates [22].

One study investigated Malaysian Chinese women living in Kuala Lumpur, Malaysia (3° N) [16]. Three studies examined Chinese populations living in Hong Kong, China (22° N) [14, 21, 22] and one study investigated Chinese women in Taipei, Taiwan (25° N) [19]. One study reported from Changsha (28° N) [17], central-south of China, one from Lanzhou (36° N) [24], north-west of China, and four studies reported results from the north-east of China, i.e., two from Beijing (40° N) [18, 20] and two from Shenyang (42° N) [15, 23]. Studies performed outside Asia could not be identified by the consulted electronic databases.

Of the 11 studies, five reported an association between the serum 25(OH)D concentration and BMD [14, 15, 17, 18, 21] while six studies did not observe an association [16, 19, 20, 22–24]; however, various sites for BMD measurement were used. Most studies measured the BMD at the lumbar spine, femoral neck, and total hip [14, 17, 20, 21]. Some studies measured the BMD at one or two of these three sites [15, 16, 19], or some of these sites were combined with the BMD of the trochanter [21, 23], or different regions in the hip [22]. In addition, some studies measured the BMD of total body [18], or of the calcaneus [24]. Finally, one study used size-adjusted BMC rather than BMD because of the comparison in bone health between Chinese people living in China and Caucasian people living in the UK [23]. All studies measured the BMD by dual energy X-ray absorptiometry (DXA), except one study which measured the calcaneal density by quantitative ultrasound (QUS) [24].

From the five cross-sectional studies with a mean baseline serum 25(OH)D concentration <50 nmol/L, two studies [17, 18] showed an association between 25(OH)D and BMD, while three studies [20, 23, 24] did not. From the cross-sectional studies with a mean baseline 25(OH)D concentration >50 nmol/L, two studies [14, 21] revealed an association and three other studies [16, 19, 22] did not.

Although some studies with Caucasian populations observed a threshold serum 25(OH)D with regard to BMD [8], none of the Chinese studies reported a threshold for the association between serum 25(OH)D concentration and BMD.

**Fig. 2:**
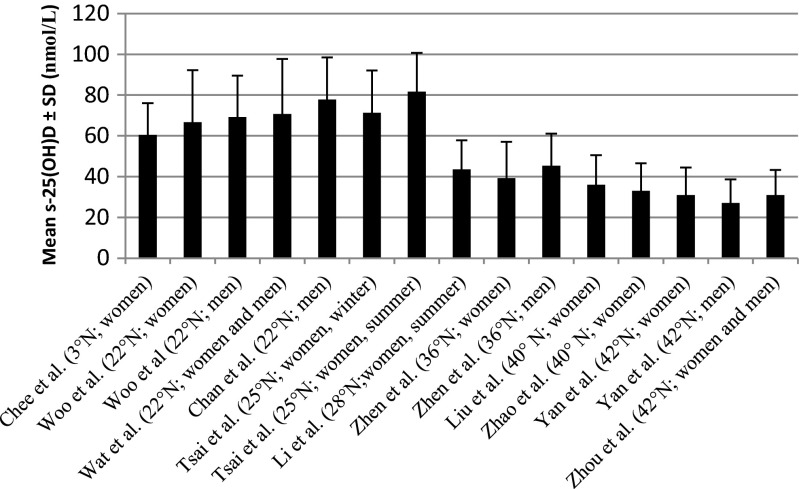
Mean serum 25(OH)D ± SD (nmol/L) of subjects in different Chinese studies according to latitude

Studies performed in north-east and north-west China showed a high prevalence of low vitamin D status [15, 18, 20, 23]. In these studies the mean serum 25(OH)D concentration of around 30 nmol/L was remarkably lower than in studies performed in south-east Asia, which reported a mean serum 25(OH)D concentration >50 nmol/L (Fig. 2).

Seasonal variation in serum 25(OH)D concentration was not observed in Hong Kong [21], whereas in Taiwan statistically lower serum 25(OH)D levels were reported when blood samples were taken in winter [19] (Fig. 2). The remaining studies included in this review did not examine seasonal variation in 25(OH)D levels.

Some of the included studies reported serum parathyroid hormone (PTH) [14, 15, 17, 21–23]. A few found a negative association between PTH and serum 25(OH)D [15, 21, 23] or between PTH and BMD [14], whereas others did not [17, 22].

In three studies [16, 20, 22], markers of bone turnover, e.g., pro-collagen Type 1 N-terminal peptide (P1NP), osteocalcin (OC), and C-telopeptide β cross link of Type 1 collagen (CTX-β), were negatively correlated with BMD at various sites. In two studies, no association was found between bone turnover markers and serum 25(OH)D [19] or PTH [23].

In woman, one study found a positive association between physical activity (number of times per week walking with a load) and BMD at the femoral neck and trochanter [22]. In another study, low physical activity was associated with serum 25(OH)D concentrations <75 nmol/L [21]. Moreover, moderate physical activity was found to be protective against vitamin D deficiency in both men and women [24].

## Discussion

The present review found no consistent relationship between the serum 25(OH)D concentration and BMD in the Chinese population: of the 11 included studies, five reported an association while six did not.

The majority of previous studies on this topic were performed in Caucasian populations. In a study among adults in the USA, higher 25(OH)D concentrations were associated with higher BMD among men and women [25]. Also, a positive association was revealed in the Longitudinal Aging Study Amsterdam (LASA) performed in the Netherlands, with a threshold of serum 25(OH)D concentration of about 50–60 nmol/L, i.e., no relation above this level [8]. In a multicenter trial among postmenopausal women with osteoporosis from 29 countries, of which 1.3 % were of Asian origin, a significant relationship was found between serum 25(OH)D levels and BMD [26]. In an Italian population of postmenopausal women a positive correlation was found between 25(OH)D concentrations and BMD at spine and hip [27], and a weak correlation was shown among ambulatory women living in Argentina [28]. A study performed in Japan among postmenopausal women reported that higher 25(OH)D concentrations were associated with a higher BMD of femoral neck [29]. In Manchester (UK), a study conducted among Pakistani women showed an association between 25(OH)D concentrations and BMD at femoral neck, total hip, lumbar spine, and distal radius BMD [30]. A modest positive correlation between the serum 25(OH)D concentration and BMD was found in White men but not in Black or Hispanic men [11]. By contrast, other studies did not show an association [31, 32].

A clear conclusion regarding the outcome of the relationship between serum 25(OH)D and BMD may be hampered by heterogeneity in, for example: inclusion and/or exclusion criteria, selection of participants, adjustments for different variables, site of BMD measurement, study size, and mean baseline serum 25(OH)D concentration.

Adjustment for confounding variables, regarded as one of the quality criteria of observational studies [33], differed between the studies (Table 1). Physical activity, which may have an effect on BMD, was described in several studies. However, no study investigated the interaction between physical activity and the relation between 25(OH)D and BMD. Two large studies, with 1000 [14] and 10,000 [24] subjects, adjusted for physical activity. Only the study with higher baseline serum 25(OH)D concentrations [14] found a cross-sectional association between serum 25(OH)D and BMD, but not in the longitudinal design. This may be due to the protective action of higher serum 25(OH)D concentrations on bone loss, since more than 50 % of the subjects had serum 25(OH)D concentrations >75 nmol/L [14]. In contrast, another smaller longitudinal study [15], with 100 subjects and a mean baseline serum 25(OH)D concentration of 30 nmol/L, did find an association between 25(OH)D and bone loss.

Of the six cross-sectional studies with a smaller study population, four of them [16, 19, 22, 23] did not show an association between 25(OH)D and BMD. However, a smaller study size may reduce the chance to detect a significant association. Three studies [16, 19, 22] had a mean baseline 25(OH)D concentration of >50 nmol/L and one study [23] of <50 nmol/L. On the other hand, by far the largest study [24], with a mean baseline 25(OH)D concentration of <50 nmol/L, also found no association. Apparently, neither the number of subjects nor the mean baseline 25(OH)D concentration seem to predict the outcome of the relationship between serum 25(OH)D and BMD.

Other variables, such as calcium supplementation and dietary calcium intake, were described in several studies [16, 19, 21, 23], but only one study adjusted for calcium supplementation [17]. Although low calcium intake may induce or aggravate vitamin D insufficiency [9], the influence of calcium is not clear from these studies.

The included studies did not explore genetic factors, although these may also influence serum 25(OH)D concentration [12, 13] and, therefore, affect the relationship between vitamin D status and BMD.

The season of 25(OH)D sampling was not described in three studies [16, 20, 22]. However, seasonal variation of serum 25(OH)D did not influence BMD values for summer and winter in Taiwan [19]. This is not remarkable when taking into account that the mean serum 25(OH)D concentration in Taiwan is >70 nmol/L, even in the winter.

One study differed from the others by measuring calcaneal density by quantitative ultrasound; however, this method may predict fracture risk as well as DXA [34].

Previous studies reported a strong relationship between the latitude of the area and vitamin D. Serum 25(OH)D levels tend to increase with decreasing latitude [26, 35]. Given the latitude of Beijing (40° N) and Shenyang (42° N) with a climate that is characterized as mild in spring and autumn, hot in summer, and very cold in winter with low exposure to sunlight, it is not surprising that serum 25(OH)D levels were lower compared with the southern regions of China, especially in late winter. Similar results for low serum 25(OH)D levels were reported in adolescent girls living in Beijing [36, 37]. However, considering Malaysia’s latitude of 3° N it is noteworthy that mean serum 25(OH)D concentrations of no more than 60 nmol/L were found in Kuala Lumpur [16]. In addition, in 50 % of the Malaysian Chinese women, serum 25(OH)D concentrations <50 nmol/L were reported. The reasons for this are unclear, but might be explained by sun-avoiding behavior, use of sunscreen, clothing habits, the hot weather, air pollution, and by limiting outdoor activities.

The relationship between vitamin D status and gender is also unclear. Serum 25(OH)D levels in Shenyang women were higher than in men [23]. In Hong Kong, male sex was associated with lower serum 25(OH)D levels [21]. By contrast, another study in Hong Kong and the study in Lanzhou observed higher serum 25(OH)D levels in men than in women [22, 24]. These differences may be due to the type of clothing, working outside, and the reluctance of women to become tanned. Previous studies were also inconclusive. A meta-analysis showed higher serum 25(OH)D levels in women than in men [38], but another review focusing on the USA revealed higher vitamin D status in men than in women [39].

A negative correlation between serum 25(OH)D and age was reported in Beijing [20], which is consistent with the decrease of serum 25(OH)D levels with aging due to insufficient sunlight exposure, a decreased functional capacity of the skin, and reductions in intestinal calcium absorption and renal function. However, in Taipei no age-related changes in serum 25(OH)D in Chinese women aged 40–72 years were found [19]. Perhaps the abundant sunshine in Taiwan may have, in part, compensated for lower 25(OH)D levels, as found in younger women [40] and in the elderly. Also, the small sample size of that study may be a reason for the lack of a correlation between serum 25(OH)D levels and age.

The present study has several limitations. First, few studies were available and most of the 11 selected studies were cross-sectional in design and susceptible to bias [33]. Moreover, most of the studies were not specifically conducted to examine a relationship between serum 25(OH)D concentrations and BMD, and not all studies used the same cut-off point to define vitamin D insufficiency and deficiency. Furthermore, studies included various bone sites for estimating BMD, and the methods used to measure BMD differed between the studies. Finally, comparison of vitamin D status may be hampered by the high variation of serum 25(OH)D measurement between laboratories [39, 41–43].

A strength of our review is that it included all studies identified by four different databases and included not only English but also Chinese language articles.

In conclusion, the evidence for a positive relationship between the serum 25(OH)D concentration and BMD in the middle-aged and older Chinese population living in Asia appears to be limited and inconsistent. Genetic factors, different lifestyle habits, including sun-avoiding behavior with the use of hats and umbrellas, the reluctance of women to become tanned, a low dietary calcium intake, and the absence of vitamin D-fortified foods may have an impact on vitamin D status and its relationship with BMD. Additional intervention studies are needed to investigate whether vitamin D supplementation can improve BMD in the Chinese population.

## Appendix A: search strategy

### PubMed

(“bone mineral density” [all fields] OR “Bone Density” [Mesh] OR “bone density” [all fields] OR BMD [tiab] OR “Bone Mineral Content” [all fields] OR “Bone Mineral Contents” [all fields] OR “bone mineral” [all fields]) AND (china [tiab] OR Chinese [tiab] OR “China” [mesh] OR “hong kong” [tiab] OR “hong kong” [mesh] OR hongkong [tw]) AND (“Vitamin D” [mesh] OR “hydroxyvitamin D” [all fields] OR “25(OH)D” [all fields] OR “vitamin D” [all fields] OR “vitamin D1” [all fields] OR “vitamin D2” [all fields] OR “vitamin D3” [all fields] OR “vitamin D4” [all fields] OR “vitamin D5” [all fields] OR vitamind [all fields] OR Cholecalciferol [all fields] OR Hydroxycholecalciferols [all fields] OR Ergocalciferols [all fields] OR Hydroxycholecalciferol [all fields] OR Ergocalciferol [all fields] OR “25-Hydroxyvitamin D 2” [all fields] OR Dihydrotachysterol [all fields] OR vitamin d status)

### EMBASE

(“bone mineral density”.ti OR *Bone Density/OR “bone density”.ti OR BMD.ti,ab OR “Bone Mineral Content”.ti OR “Bone Mineral Contents”.ti OR “bone mineral”.ti AND (exp China/ OR Chinese.ti,ab OR China.ti,ab OR “hong kong”.ti,ab OR Hong Kong/OR hongkong.ti,ab OR Chinese/) AND (exp *Vitamin D/OR (“hydroxyvitamin D” OR “25(OH)D” OR “vitamin D” OR vitamind OR Cholecalciferol OR Hydroxycholecalciferols OR Ergocalciferols OR Hydroxycholecalciferol OR Ergocalciferol OR “25-Hydroxyvitamin D 2” OR Dihydrotachysterol OR “vitamin D1” OR “vitamin D2” OR “vitamin D3” OR “vitamin D4” OR “vitamin D5” OR vitamin d status).ti)

### Web of Science

(TS = (“bone mineral density” OR Bone Density OR “bone density” OR BMD OR “Bone Mineral Content” OR “Bone Mineral Contents” OR “bone mineral”) AND TS = (China OR Chinese OR” hong kong” OR Hong Kong OR hongkong) AND TI = (“Vitamin D” OR “hydroxyvitamin D” OR “25(OH)D” OR “vitamin D” OR vitamind OR Cholecalciferol OR Hydroxycholecalciferols OR Ergocalciferols OR Hydroxycholecalciferol OR Ergocalciferol OR “25-Hydroxyvitamin D 2” OR Dihydrotachysterol OR “vitamin D1” OR “vitamin D2” OR “vitamin D3” OR “vitamin D4” OR “vitamin D5” OR vitamin d status) OR (TS = (“bone mineral density” OR Bone Density OR “bone density” OR BMD OR “Bone Mineral Content” OR “Bone Mineral Contents” OR “bone mineral”) AND TI = (China OR Chinese OR “hong kong” OR Hong Kong OR hongkong) AND TS = (“Vitamin D” OR “hydroxyvitamin D” OR “25(OH)D” OR “vitamin D” OR vitamind OR Cholecalciferol OR Hydroxycholecalciferols OR Ergocalciferols OR Hydroxycholecalciferol OR Ergocalciferol OR “25-Hydroxyvitamin D 2” OR Dihydrotachysterol OR “vitamin D1” OR “vitamin D2” OR “vitamin D3” OR “vitamin D4” OR “vitamin D5” OR vitamin d status) OR (TI = (“bone mineral density” OR Bone Density OR “bone density” OR BMD OR “Bone Mineral Content” OR “Bone Mineral Contents” OR “bone mineral”) AND TS = (China OR Chinese OR “hong kong” OR Hong Kong OR hongkong) AND TS = (“Vitamin D” OR “hydroxyvitamin D” OR “25(OH)D” OR “vitamin D” OR vitamind OR Cholecalciferol OR Hydroxycholecalciferols OR Ergocalciferols OR Hydroxycholecalciferol OR Ergocalciferol OR “25-Hydroxyvitamin D 2” OR Dihydrotachysterol OR “vitamin D1” OR “vitamin D2” OR “vitamin D3” OR “vitamin D4” OR “vitamin D5” OR vitamin d status)

### Cochrane Library

(“bone mineral density” OR Bone Density OR “bone density” OR BMD OR “Bone Mineral Content” OR “Bone Mineral Contents” OR “bone mineral”) AND (China OR Chinese OR “hong kong” OR Hong Kong OR hongkong) AND (“Vitamin D” OR “hydroxyvitamin D” OR “vitamin D” OR vitamind OR Cholecalciferol OR Hydroxycholecalciferols OR Ergocalciferols OR Hydroxycholecalciferol OR Ergocalciferol OR “25-Hydroxyvitamin D 2” OR Dihydrotachysterol OR “vitamin D1” OR “vitamin D2” OR “vitamin D3” OR “vitamin D4” OR “vitamin D5” OR vitamin d status)
